# Radiolabelled somatostatin receptor antagonist versus agonist for peptide receptor radionuclide therapy in patients with metastatic pheochromocytoma or paraganglioma: a retrospective pilot study

**DOI:** 10.1007/s00259-026-08122-8

**Published:** 2026-08-12

**Authors:** Felix Schmidt, Sofia-Maria Lider, Lisa McDougall, Christopher Eigler, Peter Bernhardt, Abdulrafee Mushaweh, Andreas Bauman, Melpomeni Fani, Emanuel Christ, Damian Wild

**Affiliations:** 1 Clinic for Radiology and Nuclear Medicine, University Hospital Basel, Petersgraben 4, Basel, CH-4031 Switzerland; 2 “Ana Aslan” National Institute of Geriatrics and Gerontology, Bucharest, Romania; 3 Department of Medical Radiation Sciences, Institution of Clinical Science, University of Gothenburg, Gothenburg, Sweden; 4 ENETS Center of Excellence for Neuroendocrine and Endocrine Tumours, University Hospital Basel, Basel, Switzerland; 5 Department of Endocrinology, University Hospital Basel, Basel, Switzerland

**Keywords:** Malignant pheochromocytoma and paraganglioma, Radioligand therapy, Somatostatin receptor, DOTA-JR11

## Abstract

**Purpose:**

Metastatic pheochromocytomas and paragangliomas (mPPGLs) are rare neuroendocrine tumours with limited therapeutic options. Peptide receptor radionuclide therapy (PRRT) using somatostatin receptor (SSTR) antagonists (e.g. [^177^Lu]Lu-DOTA-JR11) may achieve higher tumour and organ absorbed doses compared with standard SSTR agonist therapy (e.g. [^177^Lu]Lu-DOTA-TOC). This study aimed to perform an intra-patient comparison of tumour and organ dosimetry between [^177^Lu]Lu-DOTA-TOC and [^177^Lu]Lu-DOTA-JR11 in patients with mPPGLs refractory to conventional therapies. Secondary endpoints included safety and exploratory clinical efficacy of [^177^Lu]Lu-DOTA-JR11.

**Methods:**

In this retrospective pilot study, 6 patients with mPPGLs received 1–2 cycles of [^177^Lu]Lu-DOTA-TOC (~ 7.4 GBq/cycle), followed by 1–2 cycles of [^177^Lu]Lu-DOTA-JR11 (2 GBq/m^2^ × body surface area) at an interval of 10–12 weeks. Endpoints included estimation of tumour and organ absorbed doses, assessment of safety according to the Common Terminology Criteria for Adverse Events (CTCAE) v5.0 and symptoms, and evaluation of progression-free survival (PFS) before and after [^177^Lu]Lu-DOTA-JR11 therapy.

**Results:**

Intra-patient comparison showed that the median tumour absorbed dose per cycle was 1.3-fold higher (range 0.9–3.5) with [^177^Lu]Lu-DOTA-JR11 than with [^177^Lu]Lu-DOTA-TOC. This was associated with longer PFS after [^177^Lu]Lu-DOTA-JR11 (12.5 months; range 6 – >20) versus PFS before inclusion (3.5 months; range 1–9). The most severe adverse event was grade 3 lymphopenia in one patient. No thrombocytopenia, neutropenia, creatinine elevation or alanine aminotransferase elevation was observed.

**Conclusion:**

[^177^Lu]Lu-DOTA-JR11 resulted in higher tumour absorbed doses than [^177^Lu]Lu-DOTA-TOC and was associated with longer post-treatment PFS than PFS before inclusion in this small cohort. Treatment was well tolerated, supporting further prospective evaluation in patients with mPPGLs.

## Introduction

Pheochromocytomas and paragangliomas (PPGLs) are rare neuroendocrine tumours originating in the adrenal medulla and in the sympathetic or parasympathetic ganglia along the nerves. They belong to the group of neuroendocrine tumours, which arise from the neural crest [1–3]. About 10% of pheochromocytomas (PHEOs) and ~ 40% of sympathetic paragangliomas (PGLs) have malignant potential that can lead to metastasis. The most common metastatic sites are the liver, lungs, and bone [3, 4].

It is established that neuroendocrine tumours, including pheochromocytomas and paragangliomas, overexpress somatostatin receptors that are homogeneously distributed on the surface of the neoplastic cells [5–8]. Over the last decade, the theranostic use of radionuclides for diagnostic and therapeutic applications in patients with metastatic neuroendocrine tumours has increased [9–11]. In 2006, Ginj et al. were the first to raise the idea that radiolabelled somatostatin receptor (SSTR) antagonists may have an additional therapeutic benefit in these patients, possibly because of an increased number of receptor binding sites compared with SSTR agonists [12]. To date, somatostatin receptor subtype 2 (SSTR2) antagonists [^177^Lu]Lu-DOTA-JR11 (^177^Lu-satoreotide tetraxetan) and [^177^Lu]Lu-DOTA-LM3 have been shown to have higher tumour binding in human tumour specimens and to be associated with higher tumour doses and a larger number of DNA double-strand breaks compared with SSTR agonists [13]. In the first clinical trial, including 4 patients with advanced metastatic neuroendocrine tumours (NETs), patients treated with [^177^Lu]Lu-DOTA-JR11 had a 3.5-fold higher median tumour dose than those treated with [^177^Lu]Lu-DOTA-TATE [14].

For patients with metastatic PPGLs (mPPGLs), data on the effect of PRRT are scarce. To our knowledge, no clinical study has directly compared SSTR2 agonist and antagonist PRRT dosimetry within the same patients with mPPGLs. We therefore aimed to evaluate the efficacy and safety of [^177^Lu]Lu-DOTA-JR11 in patients with progressive metastatic PPGLs.

The main objective of this study was to compare tumour and organ dosimetry of [^177^Lu]Lu-DOTA-JR11 with the standard compound [^177^Lu]Lu-DOTA-TOC (SSTR2 agonist) in patients with progressive metastatic PPGLs. Secondary objectives of this study were to evaluate the efficacy and safety of [^177^Lu]Lu-DOTA-JR11 therapy.

## Materials and methods

### Study design and patients

**Fig. 1:**
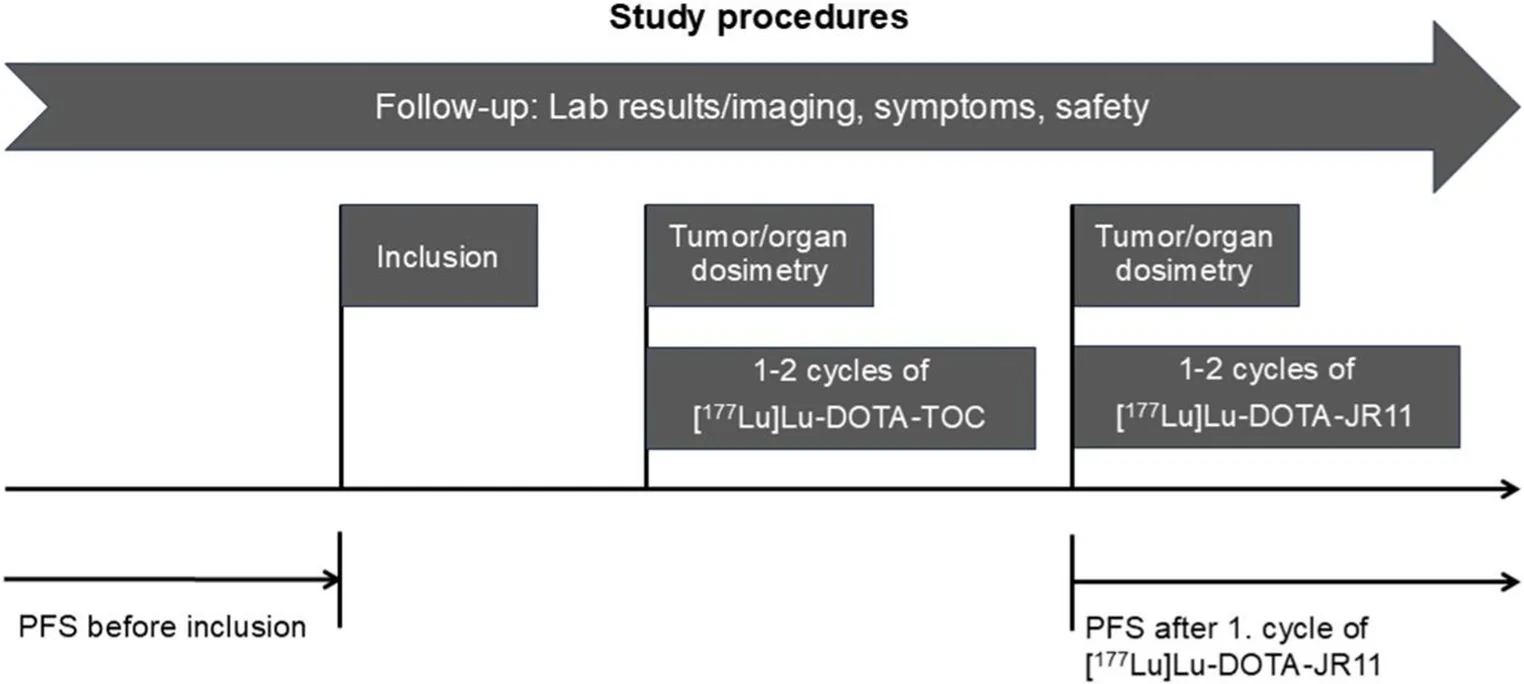
Study procedures

Patients were consecutively included in this retrospective, compassionate-use, open-label, single-centre, single-arm, intra-patient comparison study. The main inclusion criteria were histologically and imaging-confirmed metastatic PHEOs or PGLs, as defined by WHO, with radiologically progressive disease according to RECIST 1.1 and non-response to conventional treatment, measurable tumours in three dimensions, confirmed SSTR-positive disease on [^68^Ga]Ga-DOTA-TOC/-TATE PET/CT and indication for treatment with [^177^Lu]Lu-DOTA-TOC and [^177^Lu]Lu-DOTA-JR11 as assessed by the local multidisciplinary neuroendocrine tumour board. The main exclusion criteria were extensive radiotherapy involving bone marrow within the 3 months before inclusion in the study, chemotherapy within the 2 months before inclusion and Eastern Cooperative Oncology Group (ECOG) performance status > 2. The Ethics Committee of Northwest and Central Switzerland approved the data collection and evaluation (2024–02047), and all patients provided signed, written informed consent. The study procedures are shown in Fig. 1.

### Preparation of radiotracers, SPECT/CT imaging and therapy protocol

DOTA-JR11 and DOTA-TOC were synthesised according to good manufacturing practice by piCHEM GmbH (Austria) and Bachem AG (Switzerland), respectively. [^177^Lu]Lu-DOTA-JR11 was produced on an automated synthesis module (Pharmtracer, Eckert & Ziegler, Germany). Briefly, 300 µg DOTA-JR11 was dissolved in sodium acetate/ascorbic acid buffer (pH 4.5) and reacted with 4–6 GBq no-carrier-added ^177^LuCl3 (EndolucinBeta, ITM, Germany) at 83 °C for 20 min, followed by C18 solid-phase extraction. The final product was formulated in physiological saline solution containing ascorbic acid as radioprotectant, Ca-DTPA as radioisotope scavenger and ethanol as excipient. Radiochemical purity was assessed by radio-HPLC and found to be ≥ 95%. Incorporation yield was measured by radio-TLC with levels of unbound ^177^Lu ≤ 0.2%.

[^177^Lu]Lu-DOTA-TOC was produced in a kit-labelling procedure by adding 240 µg of DOTA-TOC dissolved in sodium ascorbate buffer (pH 5) to a vial containing 8 GBq no-carrier-added ^177^LuCl3, followed by heating at 95 °C for 30 min. The final product was formulated in physiological saline solution containing Ca-DTPA as radioisotope scavenger. Radiochemical purity was assessed by radio-HPLC and found to be ≥ 95%. Incorporation yield was measured by radio-TLC with levels of unbound ^177^Lu ≤ 0.5%.

To prevent potential nephrotoxicity associated with radiolabelled peptide therapy, a kidney protection protocol was used. A continuous intravenous infusion of 1000 mL physiological NaCl solution containing 20.0 mg/mL lysine and 20.7 mg/mL arginine was administered over 4 h to ensure effective nephroprotection [15]. This infusion was initiated at least 1 h before each radioligand administration and continued throughout the radiopharmaceutical infusion.

For the first cycle, [^177^Lu]Lu-DOTA-TOC was administered as a bolus infusion over 1 min with an activity of 5.6–7.4 GBq. About 10 weeks later [^177^Lu]Lu-DOTA-JR11 was infused intravenously over 2 h at a dose of 2 GBq/m^2^ × body surface area. This sequence was chosen to allow an intra-patient dosimetry comparison, with [^177^Lu]Lu-DOTA-TOC serving as the established SSTR agonist reference compound and [^177^Lu]Lu-DOTA-JR11 was administered 10–12 weeks later to limit major changes in tumour burden between the two dosimetry assessments. The number of cycles was not fixed prospectively but reflected individual multidisciplinary tumour board decisions based on disease behaviour, previous treatments, clinical condition, dosimetry and tolerance.

Quantitative single-photon emission computed tomography/computed tomography (SPECT/CT) imaging was performed at approximately 24, 48 and 168 h after each radiopharmaceutical injection. All imaging was conducted using a Symbia Intevo 16 system (Siemens Healthineers), equipped with a medium-energy, low-penetration collimator, as previously described by Eigler et al. [16].

### Dosimetry

The dosimetry method used in this study was based on the approach described by Eigler et al. [16], with modifications for the specific requirements of this investigation. Whenever feasible, at least two tumour lesions per patient were analysed. Lesions were selected if they could be clearly delineated on CT and quantitatively assessed on post-therapy SPECT/CT. The same lesions were used for the intra-patient comparison of [^177^Lu]Lu-DOTA-TOC and [^177^Lu]Lu-DOTA-JR11. Lesions affected by relevant artefacts or showing substantial overlap with adjacent physiological uptake were excluded from the quantitative analysis.

Kidney and spleen volumetry was performed using an automated AI-based segmentation tool. Lesion and organ uptake were derived from volumes of interest (VOIs) on the nuclear medicine dataset. The number of disintegrations and absorbed doses were calculated using OLINDA 1.0. For kidneys, liver, and spleen, the phantom organ weights were adjusted to the individual patient organ weights. The absorbed dose for metastatic pheochromocytoma or paraganglioma lesions was estimated using the sphere model implemented in OLINDA 1.0. Red marrow activity was determined by delineating volumes of interest in each vertebral body from Th2 to L5 at all imaging time points. The absorbed dose to the red marrow was then calculated by multiplying the time-integrated activity concentration by the absorbed energy per decay of Lutetium-177.

### Safety and tolerability

Vital parameters (blood pressure, heart rate and oxygen saturation) were monitored every 15 min during the 2 h infusion of [^177^Lu]Lu-DOTA-JR11. A full blood count and a comprehensive metabolic panel were performed on the day of each therapy cycle as well as 2, 4, and 6 weeks thereafter.

Kidney and liver function, including creatinine, alanine aminotransferase (ALT) and hypoalbuminaemia, and haematological dysfunctions (anaemia, lymphopenia, neutropenia, thrombocytopenia, or myelodysplasia) were evaluated before therapy and up to 18 months post-therapy.

Haematological dysfunctions were defined as persistent if present at more than 6 months post-therapy.

Treatment-emergent adverse events (TEAEs) such as abdominal pain, nausea, flushing, diarrhoea, constipation, hypotension, and vomiting were assessed for 6 weeks after each treatment cycle. The development of neoplasia was monitored up to 18 months post-therapy. The Common Terminology Criteria for Adverse Events (CTCAE) v5.0 were used to evaluate the grade of these adverse events.

### Efficacy

Efficacy was assessed by progression-free survival (PFS), radiological follow-up, hormonal markers where available, and symptom control. Patient follow-up began after the first cycle of [^177^Lu]Lu-DOTA-JR11 and included CT/MRI imaging, laboratory assessments, and clinical evaluation in all patients. Pretreatment PFS before study inclusion was assessed retrospectively from available imaging and clinical records. It was defined as the interval from the start of the last documented treatment line before initiation of our intra-patient comparison of [^177^Lu]Lu-DOTA-TOC and [^177^Lu]Lu-DOTA-JR11 to radiologically documented disease progression during that treatment line. Post-treatment PFS was defined as the interval from the first administration of [^177^Lu]Lu-DOTA-JR11 to documented disease progression or death from any cause. Radiological follow-up was performed using CT, MRI, and/or PET/CT at intervals of 2 to 6 months after each treatment cycle. Disease progression was assessed according to RECIST 1.1 on cross-sectional imaging; clinical or biochemical findings alone were not used to define progression. Symptom control was evaluated retrospectively through review of clinical records for up to 18 months after initiation of therapy.

### Statistical analysis

Statistical analyses were conducted using IBM SPSS Statistics for Windows, version 25.0 (IBM Corp., Armonk, NY, USA). Data are presented as medians (range), unless otherwise stated. Median progression-free survival (PFS) was estimated using the Kaplan–Meier method. PFS before and after [^177^Lu]Lu-DOTA-JR11 treatment in the same patients was compared using the Wilcoxon signed-rank test. Statistical significance was defined as a p value < 0.05. Patient numbers are given in chronological order.

## Results

### Patient characteristics and therapy regimen

Six patients with metastatic PPGLs (mPPGLs) were included in this study (four females), with a median age of 38 years (range 25–65) at tumour diagnosis and a median age of 57 years (range 45–75) at the time of [^177^Lu]Lu-DOTA-JR11 treatment. Median follow-up was 28 months (range 18–48) after initiation of [^177^Lu]Lu-DOTA-JR11 therapy. Demographic and clinical characteristics, treatment cycles, administered activities and outcomes are summarised in Table 1. Three of six patients had hereditary disease: two had germline SDHB variants and one had a germline VHL variant (Table 1).

**Table 1:** Summary of patient characteristics and previous conventional treatments

Gender		Patient 1	Patient 2	Patient 3	Patient 4	Patient 5	Patient 6
		Male	Female	Female	Male	Female	Female
Age at diagnosis (years)		39	28	65	37	65	25
Age at start of [^177^Lu]Lu-DOTA-TOC / [^177^Lu]Lu-DOTA-JR11 therapy (years)		58	56	75	45	67	51
ECOG: Before treatment After treatment		1 1	0–1 0–1	0 0	0 0	0 0	1 1
Genetic status		VHL (germline)	Not available	SDHB c.193 C > T (p.Leu65Phe) (germline)	SDHB c.338_339delinsAT (p.Cys113Tyr) (germline)	Not available	Sporadic
Duration of metastatic disease before 1st cycle of [^177^Lu]Lu-DOTA-JR11		19 years	20 years	1 month	6.4 years	7 months	12 years
Follow-up duration after 1st cycle of [^177^Lu]Lu-DOTA-JR11 (months)		35	48	25	31	18	24
Primary tumour localisation		PHEO-right	PGL-glomus caroticum	PGL-glomus caroticum	PHEO-right	PHEO-left	PGL-cervical
Tumour secretion pattern		Noradrenergic	Non-functional	Non-functional	Noradrenergic	Noradrenergic	Non-functional
Synchronous/metachronous metastases*		M	M	M	S	M	M
Metastatic sites		Lung, bone, lymph nodes	Lung, bone, lymph nodes, meningeal	Lung, bone	Liver, bone, lymph nodes	Pancreas, lymph nodes, retroperitoneal	Lung, bone
Ki-67 index		-	-	30%	30%	7%	-
Other malignant diseases		None	None	Cured breast cancer	None	None	None
Prior treatments		Radiotherapy, ^131^I-MIBG, [^177^Lu]Lu-DOTA-TOC, Sunitinib, CVD, Temozolomide, Surgery	Surgery, Radiotherapy, [^177^Lu]Lu-DOTA-TOC	Embolisation, Radiotherapy	Radiotherapy, CVD, Temozolomide, Everolimus, [^177^Lu]Lu-DOTA-TOC, Surgery	Extensive abdominal surgeries	Surgery, Sunitinib, Radiotherapy
Last documented treatment line before intra-patient comparison		Temozolomide	[^177^Lu]Lu-DOTA-TOC	Embolisation	[^177^Lu]Lu-DOTA-TOC	Extensive abdominal surgery	Surgery
Treatment between therapy cycles		-	-	-	Sunitinib Radiotherapy	-	-
[^177^Lu]Lu-DOTA-TOC (before combination therapy)		4 cycles: total 29.6 GBq	6 cycles: total 44.1 GBq	-	7 cycles: total 46.4 GBq	-	-
[^177^Lu]Lu-DOTA-JR11 + [^177^Lu]Lu-DOTA-TOC	[^177^Lu]Lu-DOTA-TOC	1 cycle: 7.4 GBq	1 cycle: 7.4 GBq	1 cycle: 7.2 GBq	2 cycles: total 11.1 GBq	1 cycle: 7.4 GBq	2 cycles: total 14.7 GBq
	Interval between therapies (weeks)	10	12	10	11	10	11
	[^177^Lu]Lu-DOTA-JR11	1 cycle: 4.3 GBq	1 cycle: 3 GBq	1 cycle: 3.2 GBq	2 cycles: total 5.9 GBq	1 cycle: 2.8 GBq	1 cycle: 4 GBq

Abbreviations: *CVD* Cyclophosphamide, Vincristine, Dacarbazine, *MIBG* metaiodobenzylguanidine, *PHEO* pheochromocytoma, *PGL* paraganglioma, *S synchronous metastases, M metachronous metastases, ECOG Eastern Cooperative Oncology Group, *SDHB* succinate dehydrogenase complex iron-sulfur subunit B, *VHL* von Hippel–Lindau

Within the intra-patient comparison protocol, four patients received one cycle and two patients received two cycles of [^177^Lu]Lu-DOTA-TOC. The median cumulative administered activity of [^177^Lu]Lu-DOTA-TOC was 7.4 GBq (range 7.2–14.7 GBq), corresponding to approximately 7.4 GBq per cycle. [^177^Lu]Lu-DOTA-JR11 was administered 10–12 weeks after [^177^Lu]Lu-DOTA-TOC. Five patients received one cycle and one patient received two cycles of [^177^Lu]Lu-DOTA-JR11. The median cumulative administered activity of [^177^Lu]Lu-DOTA-JR11 was 3.6 GBq (range 2.8–5.9 GBq), with administered activities per cycle ranging from 2.8 to 4.3 GBq. This variability reflects the retrospective compassionate-use setting rather than a fixed prospective treatment protocol.

### Dosimetry

Table 2 summarises the results of tumour and red marrow absorbed dose estimations, as well as tumour-to-bone marrow and tumour-to-kidney dose ratios, for five of the six patients. Dosimetry could not be performed in one patient due to incomplete quantitative imaging. In addition, red marrow dose could not be calculated for one patient because of disseminated spinal bone metastases.

**Table 2:** Dosimetry

Patient No.	[^177^Lu]Lu-DOTA-TOC				[^177^Lu]Lu-DOTA-JR11				[^177^Lu]Lu-DOTA-JR11/ [^177^Lu]Lu-DOTA-TOC Tumour dose Ratio
	Tumour Dose (Gy/cycle)	Red marrow Dose (Gy/cycle)	T/BM Ratio	T/K Ratio	Tumour Dose (Gy/cycle)	Red marrow Dose (Gy/cycle)	T/BM Ratio	T/K Ratio	
1	1.3	0.20	6	0.5	1.1	0.54	2	0.2	0.9
2	3.7	0.21	18	0.6	4.7	0.28	17	0.9	1.3
3	10.7	0.12	89	2.6	11.5	0.21	55	3.4	1.1
4	NA	NA	NA	NA	NA	NA	NA	NA	NA
5	3.5	0.25	14	0.7	12.1	0.38	32	2.7	3.5
6	5.5	NA	NA	0.7	7.9	NA	NA	1.3	1.4
Median	3.7	0.21	16	0.7	7.9	0.33	24	1.3	1.3
Range	1.3–10.7	0.12–0.25	6–89	0.5–2.6	1.1–12.1	0.21–0.54	2–55	0.2–3.4	0.9–3.5

Values are shown per cycle

Abbreviations: *NA* not available, *T/BM* tumour-to-bone marrow, *T/K *tumour-to-kidney. For Patient 6, T/BM was not available because red marrow dose was not available

The median tumour dose was 1.3-fold higher (range, 0.9–3.5) with [^177^Lu]Lu-DOTA-JR11 than with [^177^Lu]Lu-DOTA-TOC. Median tumour-to-kidney and tumour-to-spleen dose ratios were also more favourable with [^177^Lu]Lu-DOTA-JR11, being 1.5-fold (range, 0.4–3.9) and 2.1-fold (range, 1.9–2.3) higher than for [^177^Lu]Lu-DOTA-TOC, respectively. The median tumour-to-bone marrow dose ratio, available for four patients, was similar for both compounds.

### Efficacy

**Fig. 2:**
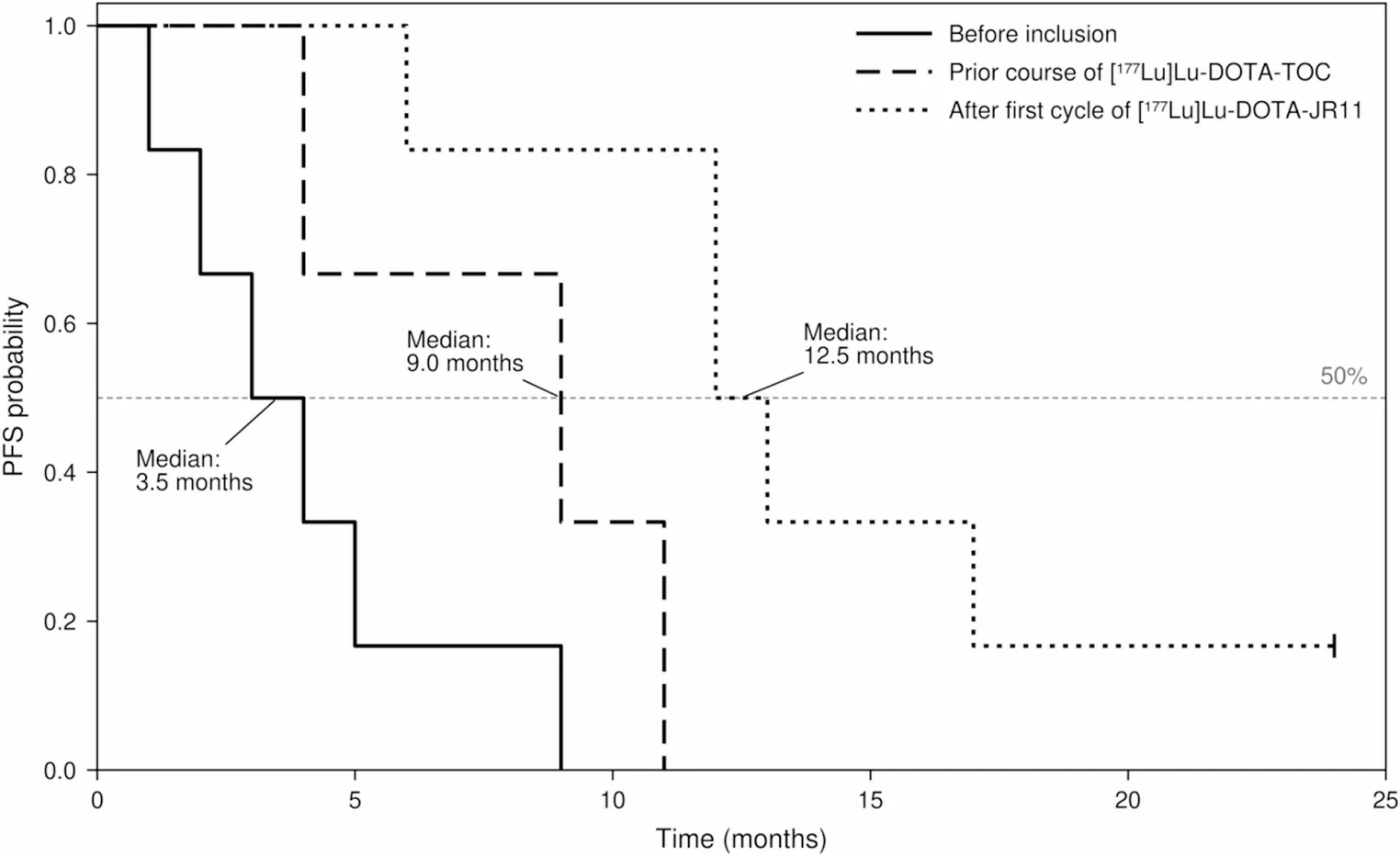
Kaplan–Meier analysis demonstrated prolonged progression-free survival (PFS) following [^177^Lu]Lu-DOTA-JR11 therapy compared with the period before study inclusion, with median PFS values of 12.5 and 3.5 months, respectively. Furthermore, the median PFS achieved during the prior course of [^177^Lu]Lu-DOTA-TOC therapy before study inclusion was 9.0 months

**Fig. 3:**
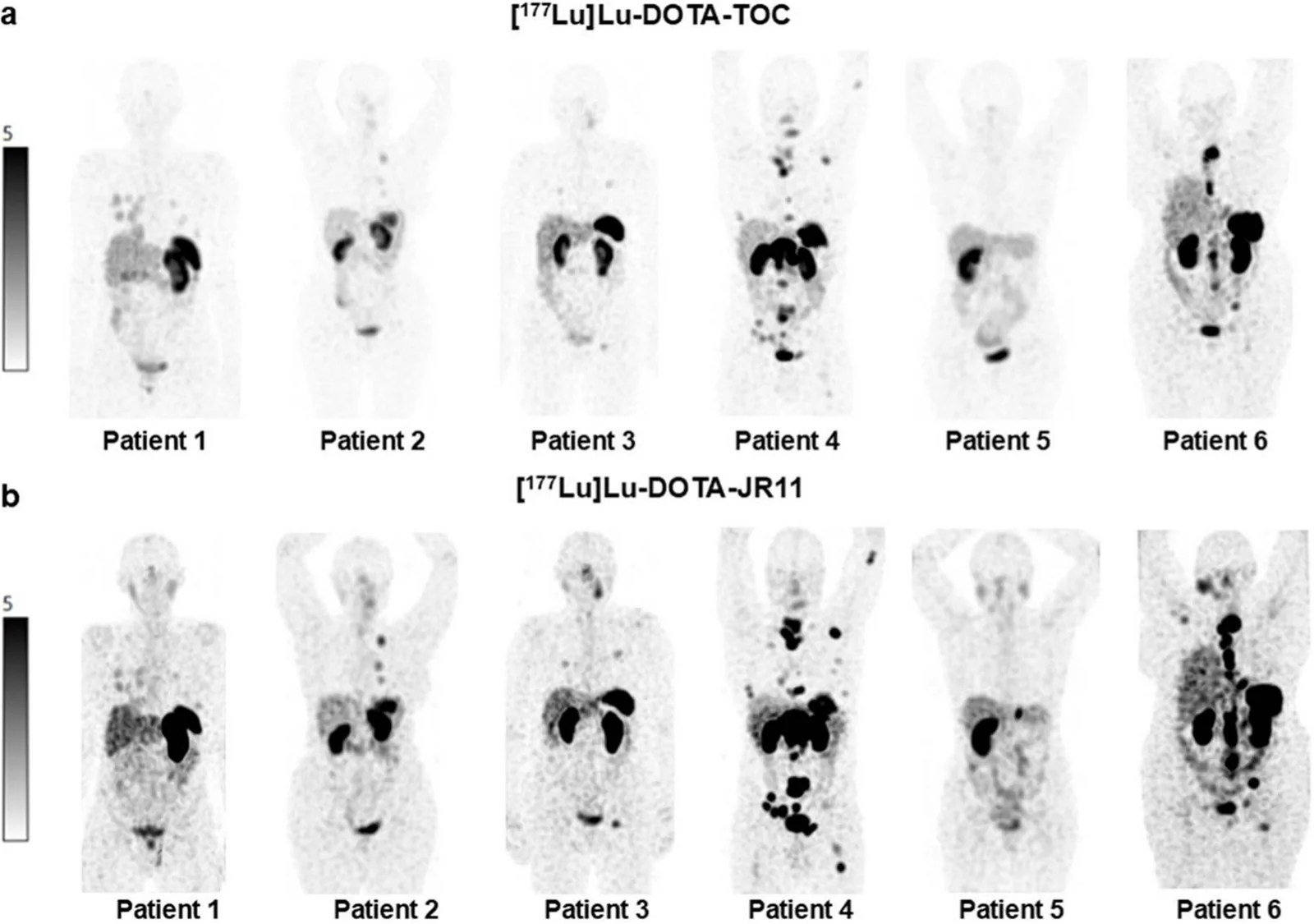
Post-treatment maximum-intensity projection images of quantitative SPECT acquired 24 h after injection of [^177^Lu]Lu-DOTA-TOC (a) and [^177^Lu]Lu-DOTA-JR11 (b) in the same patients, 10–12 weeks apart. The standardised uptake value (SUV) window threshold was set to 5 for all images, and scales indicate SUV values. In all patients, tumour uptake was visually more pronounced with [^177^Lu]Lu-DOTA-JR11 than with [^177^Lu]Lu-DOTA-TOC

In this exploratory intra-patient comparison, median (range) progression-free survival (PFS) after initiation of [^177^Lu]Lu-DOTA-JR11 therapy was 12.5 months; individual PFS values were 6, 12, 12, 13, and 17 months, while one patient remained progression-free at last follow-up and was censored at > 24 months. The median PFS of the last treatment line before study inclusion was 3.5 months (range, 1–9 months) (Table 3; Fig. 2). Representative post-treatment SPECT images are shown in Fig. 3. Among the three patients who had received a prior course of [¹⁷⁷Lu]Lu-DOTA-TOC before the intra-patient comparison, PFS after this prior course was 11, 9, and 4 months, respectively, corresponding to a median PFS of 9 months (Fig. 2). Two of three patients with functional tumours were free of adrenergic symptoms after treatment. Patient 4 received sunitinib and radiotherapy between PRRT cycles because of clinically relevant progression in the setting of high-volume disease with synchronous metastases and a Ki-67 index of 30%. Overall, three of the six patients died from tumour progression during a follow-up period of more than two years.

**Table 3:** Summary of clinical, biochemical and morphological outcomes

[^177^Lu]Lu-DOTA-TOC + [^177^Lu]Lu-DOTA-JR11	Patient 1	Patient 2	Patient 3	Patient 4	Patient 5	Patient 6
PFS after prior course of [^177^Lu]Lu-DOTA-TOC therapy (months)	11	9	NA	4	NA	NA
PFS before inclusion (months)	5	9	3	4	2	1
PFS after 1st cycle [^177^Lu]Lu-DOTA-JR11 (months)	13	17	12	6	12	> 24
PFS radio after 1st cycle [^177^Lu]Lu-DOTA-JR11 and before inclusion	2.6	1.9	4.0	1.5	6.0	> 24
Symptoms after 1st cycle [^177^Lu]Lu-DOTA-JR11	Hypertensive spells Headache	No	No	No	No	No
OS at last evaluable follow-up (months after start of 1st cycle [^177^Lu]Lu-DOTA-JR11)	OS = 35	OS = 48	OS > 25	OS > 31	OS = 18	OS > 24

Values preceded by “>” indicate censored observations

At last evaluable follow-up, three patients had died

Abbreviations: *PFS* progression-free survival, *NA* not applicable, *OS* overall survival

PFS after prior course of [^177^Lu]Lu-DOTA-TOC therapy applies only to patients who had received [^177^Lu]Lu-DOTA-TOC before the intra-patient comparison

### Safety and tolerability

All TEAEs are summarised in Table 4. No grade 4 or 5 adverse events were observed. The most frequently reported laboratory adverse events after [^177^Lu]Lu-DOTA-TOC and [^177^Lu]Lu-DOTA-JR11 were lymphopenia and anaemia (3/6 patients each), followed by hypoalbuminaemia (2/6 patients). No cases of thrombocytopenia, neutropenia, creatinine elevation or ALT elevation were recorded.

**Table 4:** Treatment-emergent adverse events (TEAEs)

[^177^Lu]Lu-DOTA-TOC + [^177^Lu]Lu-DOTA-JR11			
(*N* = 6)			
Number of patients (percent)			
TEAE	Grade 1–2	Grade 3	Reversible*
Lymphopenia	2 (33)	1† (17)	2 (67)
Anaemia	3 (50)	0	1 (33)
Hypoalbuminaemia	2 (33)	0	1 (50)
Neutropenia	0	0	
Thrombocytopenia	0	0	
ALT	0	0	
Creatinine	0	0	
Abdominal pain	2 (33)	0	2 (100)
Nausea	1 (17)	0	1 (100)
Flushing	0	0	0
Diarrhoea	0	0	0
Constipation	2 (33)	0	2 (100)
Vomiting	0	0	0
Hypotension	2 (33)	0	1 (50)
Hypertension	0	0	0
Asthenia	3 (50)	0	3 (100)
Dizziness	1 (17)	0	1 (100)
Xerostomia	1 (17)	0	1 (100)
Myelodysplastic syndrome or other neoplasia	0	0	0

Values are given as n (%). *Percentages in the “Reversible” column refer to patients with the respective TEAE. †Lymphopenia in one patient progressed from grade 2 to grade 3 but was reversible. There was no grade 4 or 5 toxicity

Other PRRT-related symptoms included grade 1 asthenia, abdominal pain, constipation, and hypotension. However, in the setting of advanced and extensive disease, the causal relationship between PRRT and non-specific symptoms such as asthenia or dizziness remains uncertain. The grade 3 lymphopenia was reversible, and most grade 2 toxicities were likewise reversible.

Up to 18 months after the first treatment cycle with [^177^Lu]Lu-DOTA-JR11, no deterioration in renal function was observed, and there was no evidence of myelodysplastic syndrome or secondary malignancy.

## Discussion

To our knowledge, this is the first study to evaluate the efficacy (progression-free survival and symptom control) of PRRT with the SSTR2 antagonist [^177^Lu]Lu-DOTA-JR11, and to compare tumour and organ absorbed doses with standard PRRT ([^177^Lu]Lu-DOTA-TOC) in the same patients with mPPGLs refractory to conventional therapies.

The main findings of this study are as follows. First, the tumour absorbed dose per cycle was 1.3-fold (range: 0.9–3.5) higher with [^177^Lu]Lu-DOTA-JR11 than with [^177^Lu]Lu-DOTA-TOC. At the same time, the absorbed doses per cycle of [^177^Lu]Lu-DOTA-JR11 and [^177^Lu]Lu-DOTA-TOC were comparable in dose-limiting organs, such as bone marrow and kidneys. Second, median PFS after [^177^Lu]Lu-DOTA-JR11 was 12.5 months (individual PFS values were 6, 12, 12, 13, and 17 months, with one patient censored at > 24 months), compared with pretreatment PFS of 3.5 months (range: 1–9). This exploratory efficacy signal must be interpreted cautiously, because all patients had received [^177^Lu]Lu-DOTA-TOC shortly before [^177^Lu]Lu-DOTA-JR11 and a carry-over effect cannot be excluded. Third, catecholamine-related symptoms were well controlled following [^177^Lu]Lu-DOTA-JR11 treatment. The single non-responding patient had an aggressive, long-standing disease with a rapidly progressive course. Fourth, treatment with a median cumulative [^177^Lu]Lu-DOTA-JR11 activity of 3.6 GBq was safe and well tolerated.

The higher tumour absorbed dose observed with [^177^Lu]Lu-DOTA-JR11 may be clinically relevant, although efficacy cannot be attributed to [^177^Lu]Lu-DOTA-JR11 alone in this retrospective sequence. In our cohort, the median PFS was 12.5 months, compared with 32 months (95% CI, 21–42) in a 2023 meta-analysis of 330 patients with metastatic or inoperable PPGLs treated with PRRT [17] and 19.9 months in a prospective phase II study of 36 patients with RECIST 1.1-defined disease progression within 12 months who received four cycles of [^177^Lu]Lu-DOTA-TATE (7.4 GBq per cycle) [18]. In this heavily pretreated cohort of patients with metastatic PPGL (11/36 previously treated with chemotherapy and/or tyrosine kinase inhibitors), the best RECIST response occurred as late as 30 months after initiation of [^177^Lu]Lu-DOTA-TATE, with an average time to best response of 11 months [18]. However, these comparisons should be interpreted with caution, as the present cohort was small, heavily pretreated, refractory to conventional therapies, and received only a limited number of therapy cycles.

Therefore, the main value of the present study is the intra-patient dosimetry comparison, whereas the clinical efficacy findings should be regarded as hypothesis-generating.

The results of this retrospective pilot study suggest that PRRT with [^177^Lu]Lu-DOTA-JR11 may be effective in patients with advanced mPPGLs. At the same time, bone marrow toxicity appears to represent a potential limitation for the broader clinical use of [^177^Lu]Lu-DOTA-JR11 [19]. Notably, both haematological and overall toxicity observed in our cohort were lower than those reported in prior studies evaluating [^177^Lu]Lu-DOTA-JR11 [19]. This discrepancy is likely attributable to the individualised treatment approach and small number of treatment cycles applied in our study, in which administered activity was adjusted according to body surface area (2 GBq/m^2^) [16]. Using this strategy, the injected activity per cycle (range: 2.8–4.3 GBq) was lower than that reported in studies employing fixed-dose regimens (~ 7 or ~ 4.5 GBq per cycle) [19, 20]. Given the established relationship between administered activity and absorbed bone marrow dose, the reduced activity in our cohort likely translated into lower bone marrow irradiation and, accordingly, reduced haematotoxicity. Renal toxicity appears to be unlikely following treatment with [^177^Lu]Lu-DOTA-JR11, given the low median absorbed kidney dose per cycle of 5.0 Gy (range: 3.3–6.3 Gy) and a median tumour-to-kidney absorbed dose ratio that is approximately 1.5-fold higher than that reported for standard therapy with [^177^Lu]Lu-DOTA-TOC.

This study has several limitations. First, the small sample size limits the generalisability of the findings. Given the small sample size and the presence of one censored PFS observation after treatment with [^177^Lu]Lu-DOTA-JR11, no formal paired statistical comparison was performed. PFS before and after treatment is therefore reported descriptively. Second, all patients received at least one cycle of [^177^Lu]Lu-DOTA-TOC (two patients received two cycles) 10–12 weeks prior to initiation of [^177^Lu]Lu-DOTA-JR11. This may have led to an overestimation of PFS for [^177^Lu]Lu-DOTA-JR11 due to a carry-over effect from the immediately preceding [^177^Lu]Lu-DOTA-TOC therapy and limits attribution of efficacy to [^177^Lu]Lu-DOTA-JR11 alone. In addition, patient 4 received sunitinib and radiotherapy between PRRT cycles, which further limits treatment attribution in this patient. Nevertheless, a median PFS of 12.5 months appears clinically relevant, particularly given the low number of administered cycles (median, one cycle [^177^Lu]Lu-DOTA-JR11). Third, this is a retrospective study with a personalised approach based on a multidisciplinary tumour board recommendation. Therefore, the study reflects a real-world approach with a personalised number of therapy cycles. Fourth, hormonal markers were not included in the analysis because they were measured inconsistently at different time points, precluding meaningful interpretation.

## Conclusion

[^177^Lu]Lu-DOTA-JR11 resulted in a 1.3-fold higher tumour absorbed dose (range 0.9–3.5) compared with [^177^Lu]Lu-DOTA-TOC in patients with mPPGLs and was associated with longer post-treatment PFS than pretreatment PFS and symptom control in this small cohort. Given the retrospective design and preceding [^177^Lu]Lu-DOTA-TOC therapy, these clinical findings should be interpreted as exploratory. The encouraging preliminary results regarding the dosimetry and tolerability warrant further evaluation in larger, prospective studies.

## Data Availability

The datasets generated and/or analysed during the current study are not publicly available due to patient privacy restrictions but are available from the corresponding author on reasonable request.
